# Individual patient variability with the application of the kidney failure risk equation in advanced chronic kidney disease

**DOI:** 10.1371/journal.pone.0198456

**Published:** 2018-06-12

**Authors:** Christopher McCudden, Ayub Akbari, Christine A. White, Mohan Biyani, Swapnil Hiremath, Pierre Antoine Brown, Navdeep Tangri, Scott Brimble, Greg Knoll, Peter G. Blake, Manish M. Sood

**Affiliations:** 1 Department of Pathology and Laboratory Medicine, University of Ottawa, Ottawa, Ontario, Canada; 2 Division of Nephrology, Department of Medicine, University of Ottawa, Ottawa, Ontario, Canada; 3 Kidney Research Centre/The Ottawa Hospital Research Institute, Ottawa, Ontario, Canada; 4 Division of Nephrology, Department of Medicine, Queen’s University, Kingston, Ontario, Canada; 5 Seven Oaks General Hospital, University of Manitoba, Winnipeg, Manitoba, Canada; 6 Division of Nephrology, Department of Medicine, McMaster University, Hamilton, Canada; 7 Western University and London Health Sciences Centre, London, Canada; Kaohsiung Medical University Hospital, TAIWAN

## Abstract

The Kidney Failure Risk Equation (KFRE) predicts the need for dialysis or transplantation using age, sex, estimated glomerular filtration rate (eGFR), and urine albumin to creatinine ratio (ACR). The eGFR and ACR have known biological and analytical variability. We examined the effect of biological and analytical variability of eGFR and ACR on the 2-year KFRE predicted kidney failure probabilities using single measure and the average of repeat measures of simulated eGFR and ACR. Previously reported values for coefficient of variation (CV) for ACR and eGFR were used to calculate day to day variability. Variation was also examined with outpatient laboratory data from patients with an eGFR between 15 and 50 mL/min/1.72 m^2^. A web application was developed to calculate and model day to day variation in risk. The biological and analytical variability related to ACR and eGFR lead to variation in the predicted probability of kidney failure. A male patient age 50, ACR 30 mg/mmol and eGFR 25, had a day to day variation in risk of 7% (KFRE point estimate: 17%, variability range 14% to 21%). The addition of inter laboratory variation due to different instrumentation increased the variability to 9% (KFRE point estimate 17%, variability range 13% to 22%). Averaging of repeated measures of eGFR and ACR significantly decreased the variability (KFRE point estimate 17%, variability range 15% to 19%). These findings were consistent when using outpatient laboratory data which showed that most patients had a KFRE 2-year risk variability of ≤ 5% (79% of patients). Approximately 13% of patients had variability from 5–10% and 8% had variability > 10%. The mean age (SD) of this cohort was 64 (15) years, 36% were females, the mean (SD) eGFR was 32 (10) ml/min/1.73m^2^ and median (IQR) ACR was 22.7 (110). Biological and analytical variation intrinsic to the eGFR and ACR may lead to a substantial degree of variability that decreases with repeat measures. Use of a web application may help physicians and patients understand individual patient’s risk variability and communicate risk (https://mccudden.shinyapps.io/kfre_app/). The web application allows the user to alter age, gender, eGFR, ACR, CV (for both eGFR and ACR) as well as units of measurements for ACR (g/mol versus mg/g).

## Introduction

The growing advanced chronic kidney disease (CKD) population is a widely recognized global health issue[1]. In patients with advanced and declining kidney function, multiple interventions including modality education and vascular access planning are required, ideally prior to the development of end stage kidney disease (ESKD) [2]. However, most patients with CKD do not progress to ESKD [3]. Thus assessment of risk of ESKD is necessary for counseling of patients and adequate advanced care planning.

A number of risk prediction tools have been developed to quantitate the risk of progression to ESKD [4–6]. The most easily applicable, recently developed with comprehensive validation is the Kidney Failure Risk Equation (KRFE) [5, 6]. In its simplest iteration, the equation requires age, sex, estimated glomerular filtration rate (eGFR) calculated by CKD-EPI formula [7], and the urine albumin to creatinine ratio (ACR) to predict need for dialysis or renal transplantation (kidney failure) over short (2-years) and longer (5 year) time horizons. The KFRE has been adopted as a population-level tool for predicting the need for renal replacement therapies [8]. Other potential roles for KFRE include determining appropriate timing of entry into a multidisciplinary clinic, vascular access referral and modality planning or transplantation [9].

Two of the four variables included in the KFRE (eGFR and ACR) are biochemical tests with known within laboratory day-to-day variability due to biological, analytical, and pre-analytical factors [10–12]. Biological variation refers to differences in serial laboratory values due to physiological changes. Biological variation results from differences in hydration status, posture, physical activity, diet, stress, time of day, and season. Analytical variation also contributes to differences in serial laboratory values and is caused by changes in reagent lot, calibration, and assay imprecision (noise). Pre-analytical variation also occurs when considering serial samples, and arises from differences in sample collection, handling, transport, and storage. Collectively, these together account for day-to-day variation observed with repeated measures in the same laboratory on different days. When patients have their testing done at different laboratories there is additional analytical variation (Inter-laboratory variation) due to differences in assay methods and instrumentation (collection, handling, and transport procedures may also vary). The variability of the kidney failure prediction using the KFRE due to day-to-day and inter- laboratory variation of eGFR and ACR in individual patients has not been assessed. Thus, we set out to determine the influence of day-to-day and inter-laboratory variability of eGFR and ACR on the calculated risk of kidney failure in individuals using the 4 variable KFRE [6] for north American population in patients with advanced CKD. This study was approved by the Ottawa Health Science Network Research Ethics Board. The requirement for informed consent was waived as this was a simulation and retrospective study.

## Materials and methods

This was a two-part study using simulated and real patient data.

### Simulated data

We used a range of values for eGFR (15–50 mL/min/1.72 m^2^ using increments of 1 mL/min/1.72 m^2^) and ACR (1–300 mg/mmol using increments of 1 mg/mmol) yielding ~10,476 simulated data points. The study outcome was the predicted risk of kidney failure as determined by the 4-variable, 2-year KFRE for North America [6]; the simplest and shortest form of the equation was chosen. KFRE was calculated assuming 50 year old male patient. We also developed a web application to allow individuals to vary age, sex, eGFR, ACR and CV (https://mccudden.shinyapps.io/kfre_app/).

We simulated the variability in the KFRE estimates using reported biological and analytical coefficient of variations (CV) (Table 1)[12, 13];

**Table 1:** Variation factors used for risk score simulations.

Variable	Day-to-day variability	Inter-assay Variation	Reduced Variation
ACR	11.3%	9.6%	3.6%
eGFR	6.6%	4.3%	4.7%*

*Based on averaging of serial results in patients with stable kidney function

The effects of pre-analytical variation were not included. The coefficient of variation is a measure of imprecision and is calculated as SD*100/mean. For ACR and eGFR testing done by the same laboratory, we used published estimates of the average day-to-day CV, which includes biological and day-to-day intra-laboratory analytical variability. The day-to-day CVs used for simulations were 11.3% for ACR and 6.6% for eGFR based on the study by Selvin et al [12]; the study by Selvin examined within-person variability in kidney measures including ACR and eGFR using NHANES data to establish differences in a large cohort of patient results between samples. For ACR and eGFR being performed at different laboratories, the inter-laboratory analytical CV was derived from proficiency testing material data [14]; proficiency testing is a form of quality assurance where numerous laboratories test unknown patient samples and variation statistics are calculated and labs scored on their performance. Differences vary with the manufacturer, but the overall average variability between instruments is 9.6% for ACR and 4.3% for eGFR [15].

All simulations and calculations were done using the statistical programming language R [16]. and the web application was developed using RShiny.

### Variation modeling and simulation

First, eGFR was varied by 6.6% at a fixed ACR of 30 mg/mmol. Second, ACR was varied by 11.3% with a fixed eGFR of 25 ml/min/1.73m^2^. Third, ACR and eGFR variability were combined at different ACRs of 3, 30, and 300 mg/mmol and continuous eGFRs from 15 to 50 ml/min/1.73m^2^. Lastly, inter-laboratory variation was included in the simulations.[14] The combined ACR + eGFR + inter-laboratory simulation aimed to estimate the maximal effect of variability from all sources on the kidney failure prediction probability calculated using the KFRE. The variation of each component was summed according to the equation:

$$\mathrm{Total}\;\mathrm{coefficient}\;\mathrm{of}\;\mathrm{variation}=\sqrt{{CV}_{1}^{2}+{CV}_{2}^{2}+\cdots \;{CV}_{n}^{2}}$$

The above equation is based on the addition of variation components according to Fraser [17], which states that when the mean of the variation components is the same, the CV may be added; accordingly, inter-and intra-laboratory CVs were selected to match to the concentrations used to report biological variation.

After simulating the potential variability of KRFE scores using single laboratory measures, we explored options to reduce the variability. It is reported that first-morning urine sample collections averaged over consecutive days yields a log ACR variation of 3.6% (Table 1) [18]. In addition, repeat determination of eGFR was modeled from the effect averaging eGFR results. Based on analysis of serial eGFR results in patients with stable renal function (see study cohort below), averaging serial results yielded a CV of 4.7%. Accordingly, 4.7% was used for simulations of reduced variation.

### Variation of kidney failure prediction in patients

#### Study cohort

Retrospective laboratory data included 2208 pairs of serial samples from 1104 patients extracted from the Ottawa Hospital Laboratory Information System. This database captures information on many laboratory measures from an urban center (catchment area 1.2 million) and included 2 years of data. eGFR was calculated by the CKD-EPI equation [7]. We included all patients above 18 years of age who had two values of eGFR and ACR performed (n = 77,401 samples). Exclusions included (n = 75,193 samples): inpatients, dialysis patients, emergency patients, multiple repeat values (more than 2) as multiple readings are more likely to be performed in unstable patients, those with missing values, or values > 3 months apart. Variation of KFRE by timing of samples is shown in S1 Fig. There was no significant difference in variation between time periods of < 10 days to time period of 10 days to 3 months (p = 0.16). Also excluded were samples with eGFR values <15 or >50 mL/min/1.72 m^2^, (patients with eGFR < 15 ml/min/1.73m^2^ are already considered as ESRD; conversely the 2-year risk of needing renal replacement therapy with eGFR > 50 ml/min/1.73m^2^ is low). All measures were performed at The Ottawa Hospital, by the Eastern Ontario Regional Laboratory Association an accredited laboratory. All patients had serum and urine creatinine measured on the Siemens Dimension Vista 1500 using a creatininase enzyme-coupled reaction (this method is IDMS traceable). Urine albumin was measured on the Siemens Dimension Vista 1500 by immune-complex mediated nephelometry (this method uses an albumin-specific antibody reaction with measurement of scattered light). The ACR was automatically calculated in the laboratory information system, dividing the urine albumin by urine creatinine to yield ACR in mg/mmol.

Two-year kidney failure probabilities by KFRE were calculated for each ACR-eGFR measurement pair; pairs consisted of serial ACR-eGFR measurements on the same patient within 3 months. Variation in kidney failure probability was determined by calculating the difference between sequential probabilities; for example, if the kidney failure probability was calculated as 10% from laboratory values at the first visit, and the next ACR/eGFR measurements two weeks later yielded a prediction of 11%, then the probability variation was 1% (absolute difference). In addition, the mean variation of different combinations of eGFR and ACR for the whole cohort was calculated.

## Results

### Variation in KFRE risk with day-to-day variability of only eGFR

**Fig 1:**
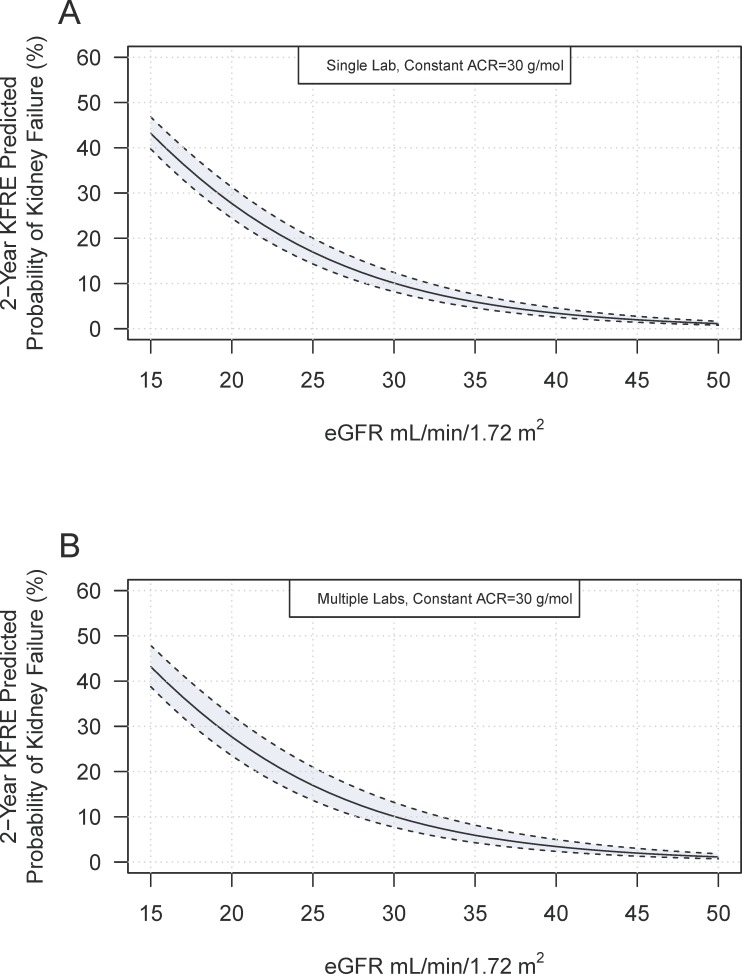
**A. Predicted KFRE variation with eGFR.** The black line represents the 2-year KFRE estimate for a 50-year old male with a fixed ACR of 30 g/mol. Upper and lower bounds (dotted lines) represent the 95% confidence interval for KFRE risk based on a biological variation in eGFR of 6.6%. **B. Predicted KFRE variation with eGFR and inter-laboratory variation.** Upper and lower bounds (dotted lines) represent the 95% confidence interval for KFRE risk based on a biological variation in eGFR of 6.6% combined with a reported inter-laboratory variation of 4.3%.

Simulation demonstrated a significant variation of risk for kidney failure by KFRE when ACR was fixed at 30 mg/mmol and assumed to have no variability. KFRE variability increased with decreasing eGFR (Fig 1A). Variability at an eGFR of 25 ml/min/1.73m^2^ was 6% (KFRE point estimate17%, variability range 14% to 20%). Simulations that also included inter-laboratory variability widened the variability of the estimates substantially (Fig 1B); for the same patient the variability increased to 8% (KFRE point estimate 17%, variability range 13% to 21%).

### Variation in KFRE risk with day-to-day variability of only ACR

**Fig 2:**
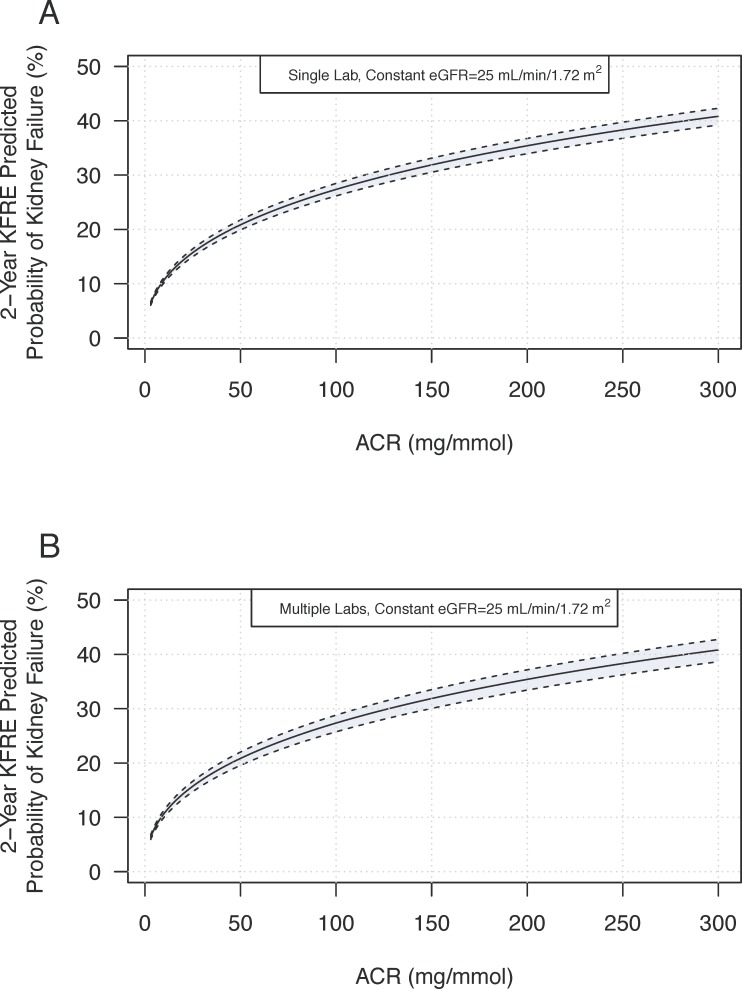
**A Predicted KFRE variation with ACR concentration**. The black line represents the 2-year KFRE estimate for a 50-year old male with a fixed eGFR of 30 mL/min/1.72 m^2^. Upper and lower bounds (dotted lines) represent the 95% confidence interval for the 2-year KFRE risk based on published ACR-concentration variation from 11.3%. **B. Predicted KFRE variation with ACR concentration in combination with inter-laboratory variation.** Upper and lower bounds (dotted lines) represent the 95% confidence interval for KFRE risk based published ACR variation combined with a reported inter-laboratory variation of 9.6%.

Similar simulations for ACR, when eGFR was fixed at 25 ml/min/1.73m^2^ and assumed to have no variability, demonstrated increased variability with rising ACR (Fig 2A). At ACR of 30 mg/mmol and eGFR of 25 ml/min/1.73m^2^, the KFRE varied by 2% (2-year KFRE point estimate 17%, variability range 16% to 18%). The addition of inter-laboratory differences widened the variability of the estimates from 2% to 3% (KFRE point estimate 17%, variability range 15% to 18%; Fig 2B).

### Variation in KFRE risk with day-to-day variability of both eGFR and ACR

**Fig 3:**
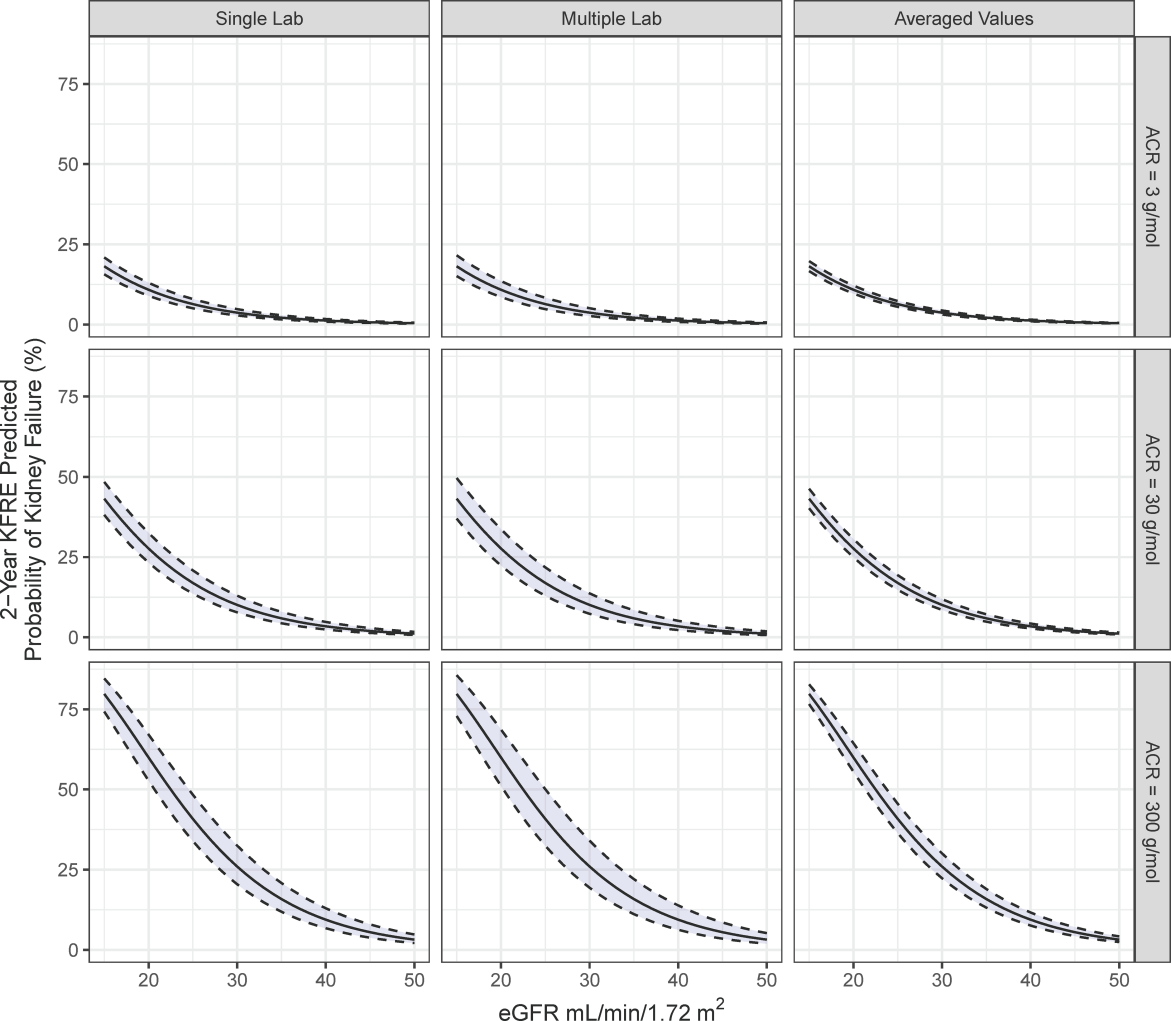
Comparison of predicted risk variability using a single laboratory, multiple laboratory with different methods, or averaging serial values. In all panels, the black lines represent the 2-year KFRE estimate for a 50-year old male at fixed ACRs of 3, 30, and 300 g/mol and the upper and lower dotted lines represent 95% confidence intervals. **Left Panels: Predicted KFRE with combined variation in eGFR and ACR analyzed by a single method. Center Panels**: Predicted KFRE with combined variation in eGFR, ACR, and laboratory method. Variation includes combined inter-laboratory variation. **Right Panels**: Predicted reduction in KFRE variation by using averages of serial eGFR and ACR measurement. Inter-laboratory variation was excluded to reflect use of a single laboratory.

Simulations revealed that the variability in KFRE risk increased when the day-to-day variability of both eGFR and ACR was combined. Simulations of combined eGFR and ACR variability are presented in Fig 3. The lower the eGFR and the higher the ACR, the higher the KFRE variability. At eGFR of 25 ml/min/1.73m^2^and ACR of 30 mg/mmol the variation was estimated at 7% (KFRE point estimate 17%, variability range 14% to 21%) Addition of inter-laboratory variation increased the variation of KFRE predicted probabilities even further (Fig 3, centre panels). For example, for same individual at an eGFR of 25 mL/min/1.72m^2^ and an ACR of 30 mg/mmol the 2-year kidney failure variation in risk increased to 9% (KFRE point estimate 17%, variability range 13% to 22%). Moreover, there were no significant differences in variability of kidney failure probability between different ages and sexes (see S1 Table).”

### Variability in KFRE risk using averaged measures of eGFR and ACR

When simulations were performed for repeated averaged values (single laboratory), the variability in risk decreased as shown in Fig 3 (right panels). At an eGFR of 25 ml/min/1.73m^2^ and ACR of 30 mg/mmol, the variability decreased from 7% to 4% (KFRE point estimate 17%, variability range 15–19%).

### Variability in KFRE risk calculated from laboratory values (cohort study)

**Fig 4:**
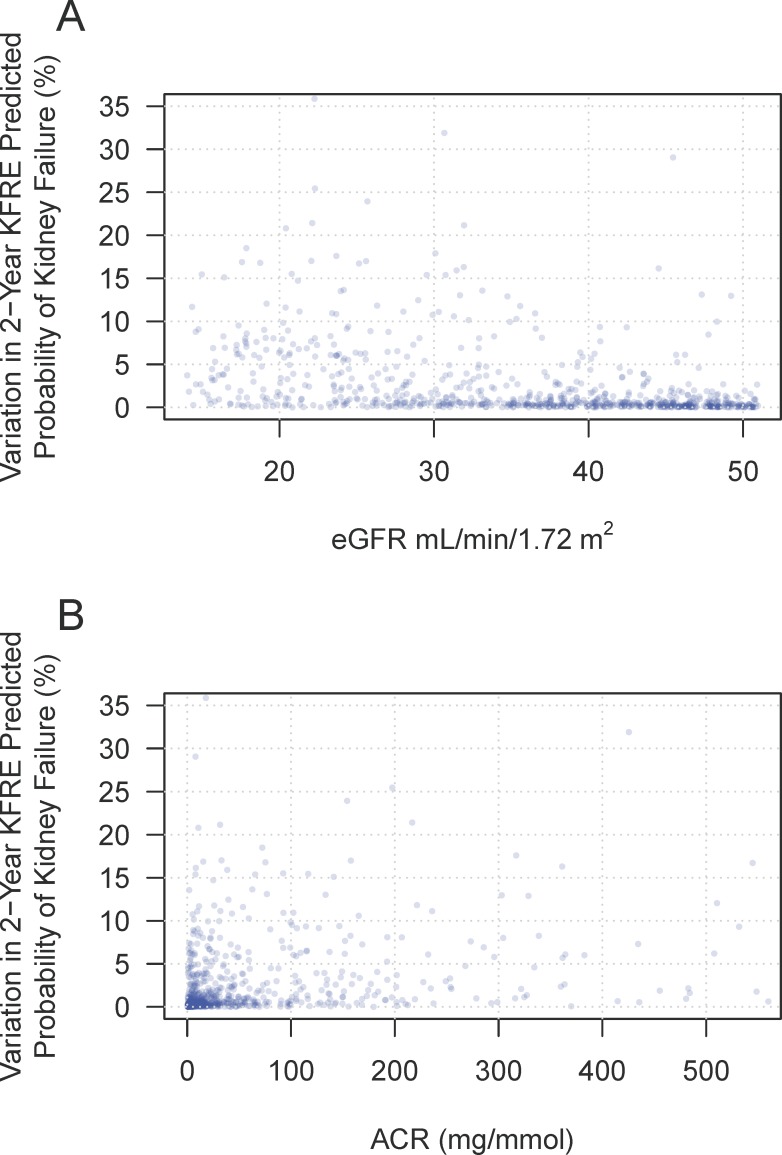
Variation in KFRE using serial eGFR and ACR from patients. **A. Variation in 2-year KFRE with eGFR in patients.** Variation was determined by calculating the difference in 2-year KFRE using serial eGFR and ACR values from a given patient. Each point represents the difference in KFRE calculated from two pairs of eGFR and ACR values for a single patient within a 3-month time period. **B. The same patient data as panel A, except showing variation in 2-year KFRE with ACR on the x-axis**.

The mean age of patients was 64 years (SD = 15) and 36% were female. The median ACR was 22.7 (IQR = 110) mg/mmol and mean eGFR was 32 (SD = 10) ml/min/1.73m^2^. Mean time between paired measures was 32 (SD = 25) days. Similar to the simulations, the variation in kidney failure probability in individual patients was eGFR and ACR-dependent. Fig 4 shows the variation in KFRE-based prediction calculated from the difference between probabilities of serial sample collections (pairs of ACR and eGFR at clinic visit).

Table 2 shows the mean variation in KFRE-based prediction calculated from the difference between probabilities of serial sample collections (pairs of ACR and eGFR at clinic visit) for different combinations of eGFR and ACR. Most patients had a KFRE 2-year risk variability of ≤ 5% (79% of patients). Approximately 13% of patients had variability from 5–10% and 8% had variability > 10%.

**Table 2:** Mean variability in KFRE risk*.

	eGFR (mL/min/1.73m^2^)			
ACR (g/mol)	15–25	25–35	35–45	45–50
**<3**	1.3% (26)	0.7% (57)	0.3% (79)	0.1% (42)
**3–30**	3.4% (131)	1.7% (159)	0.7% (185)	0.3% (135)
**30–300**	6.0% (180)	3.1% (149)	1.5% (116)	0.7% (71)
**>300**	9.0% (81)	8.2% (46)	4.8% (26)	3.4% (13)

*Values are the mean variation in 2-year KFRE risk; the number of samples in each category is shown (n)

## Discussion

To our knowledge this is the first study simulating the day-to-day variability of KFRE-based probability due to day-to-day variation of eGFR and ACR. We have shown significant variability in KFRE risk across all models, which was greatest at lower levels of eGFR and higher levels of ACR. Addition of variability due to inter-laboratory differences further increased the KFRE variability estimates. Reductions in KFRE variability were achieved using repeat averaged measures of first morning ACR and averaged eGFR. With real patient data, the majority of patients (79%) had variability in the 2-year KFRE risk of 5% or less; conversely only 8% had variability of greater than 10%.

The finding of eGFR variability is not surprising given the known variability (intra and inter-laboratory) in Cr measurement despite IDMS standardization [19, 20]. eGFR inter-laboratory variation has been reported to be as high as 20% [14]. In addition, Cr-based eGFR is also affected by several non-GFR related factors, which will contribute to biological variability. For example, dietary meat ingestion is associated with higher serum creatinine/lower eGFR whereas plant-based diets are associated with lower serum creatinine /higher eGFR measures [21, 22]. Thus the timing of a Cr measure in relation to a patient’s diet may impact their estimated risk [21, 22]. Medications such as sulphamethoxazole trimethoprim, cimetidine, ranitidine and fenofibrate may increase serum creatinine/lower eGFR and thus impact on risk score if use is not constant [23]. Further the diurnal variation in GFR should also be considered. For example a 9% GFR variation has been reported between recumbent and daytime measurements [24]. The ACR has not been standardized as yet.

The study results highlight the importance of considering risk not as a single point but rather as a risk range with expected fluctuations due to variability of the eGFR and ACR. Recognition and awareness of the day-to-day variability in kidney failure estimates should aid in conveying the risks of CKD to patients. The study results should also help inform clinical decision-making and resource allocation by healthcare providers. We have also provided an online calculator (https://mccudden.shinyapps.io/kfre_app/), which could be utilized for determining KFRE variability in patients. It calculates the risk range around the risk for single laboratory, for different laboratories and for averaged values. It also allows for change of units between conventional units and SI units. In addition the calculator allows clinicians to alter the CV for both eGFR and ACR as different populations and laboratories may have different CV. The use of repeat, serial measures should be encouraged prior to clinical intervention such as dialysis planning issues, vascular access or peritoneal dialysis catheter placement and preparation for kidney transplantation. We propose repeat measurements 3 months apart as the risk is unlikely to vary considerably in 3 months and diagnosis of CKD also requires two values 3 months apart. We also recommend that serial measures be performed in a single laboratory to reduce variability due to inter-laboratory differences in Cr and albumin measurement.

Limitations to our study should be noted. Estimates of biological and analytical variability are themselves variable but we used conservative estimates. We could not quantify the pre-analytical factors in our calculations as data are not available on how pre-analytical factors impact eGFR and ACR. Adding the pre-analytical variation of eGFR and ACR would increase the variability. The patient data represent a relatively small sample size at one institution that were having repeat measures for clinical reasons and may not have been in steady state. We would recommend that in future studies, longitudinal data from large data sets from multiple institutes should be pooled together and trends of KFRE with outcome of renal replacement therapy should be examined. The study used a fixed age, and sex, although variation in kidney failure probability was not significantly different between sexes; although KFRE risk point estimate varies with sex as well as different eGFR and ACR (see S1 Table), the variability is not significantly different at the same KFRE. To overcome this limitation, we have provided a calculator to estimate the KFRE at wide range of age and for both genders. In addition, the KFRE was validated in many populations where ACR was not measured directly but ACR was calculated from urine analysis, 24-hour urine protein and protein to creatinine ratio. Our simulations are limited to where ACR was measured directly and we are unable to comment on the variability of conversion of other measures of proteinuria to ACR. Our calculations reflect that eGFR and ACR vary independently from each other but no good data is available in this respect and this represents a future area of research.

Strengths of our study include using real patient data in addition to simulation and the results indicate our simulations are similar to real patient data. Other strengths are the use of published data for variability of ACR and eGFR, and provision of the web application. The web application is particularly useful to visualize variability in response to changing the equation parameters. It is important to consider that other tools for risk prediction and clinical decision making also have variability associated with them such as the eGFR alone and the Framingham risk score, the MELD score and others [19, 25, 26].

In summary, biological and analytical variation in eGFR and ACR values lead to variability in KFRE estimates of kidney failure. This variability may be reduced by averaging serial laboratory values and serial monitoring in a single laboratory and may be conveyed using a risk range rather than a single point value.

## Supporting information

S1 Fig Difference between serial KFRE risk (%) between time periods of 0 to 90 days. No signiciant difference was found between time periods of < 10 days and 10 days to 3 months (p = 0.16).
(PDF)

S1 Table Day-to-day variability of kidney failure risk with different ages in males and females. Note the point estimate of KFRE changes but variability not significantly different at the same KFRE.
(DOCX)
