# Supplementary figures and images for: Individual patient variability with the application of the kidney failure risk equation in advanced chronic kidney disease
Source: PLoS One. 2018 Jun 12;13(6):e0198456. doi: 10.1371/journal.pone.0198456 (PMC5997334; doi:10.1371/journal.pone.0198456)

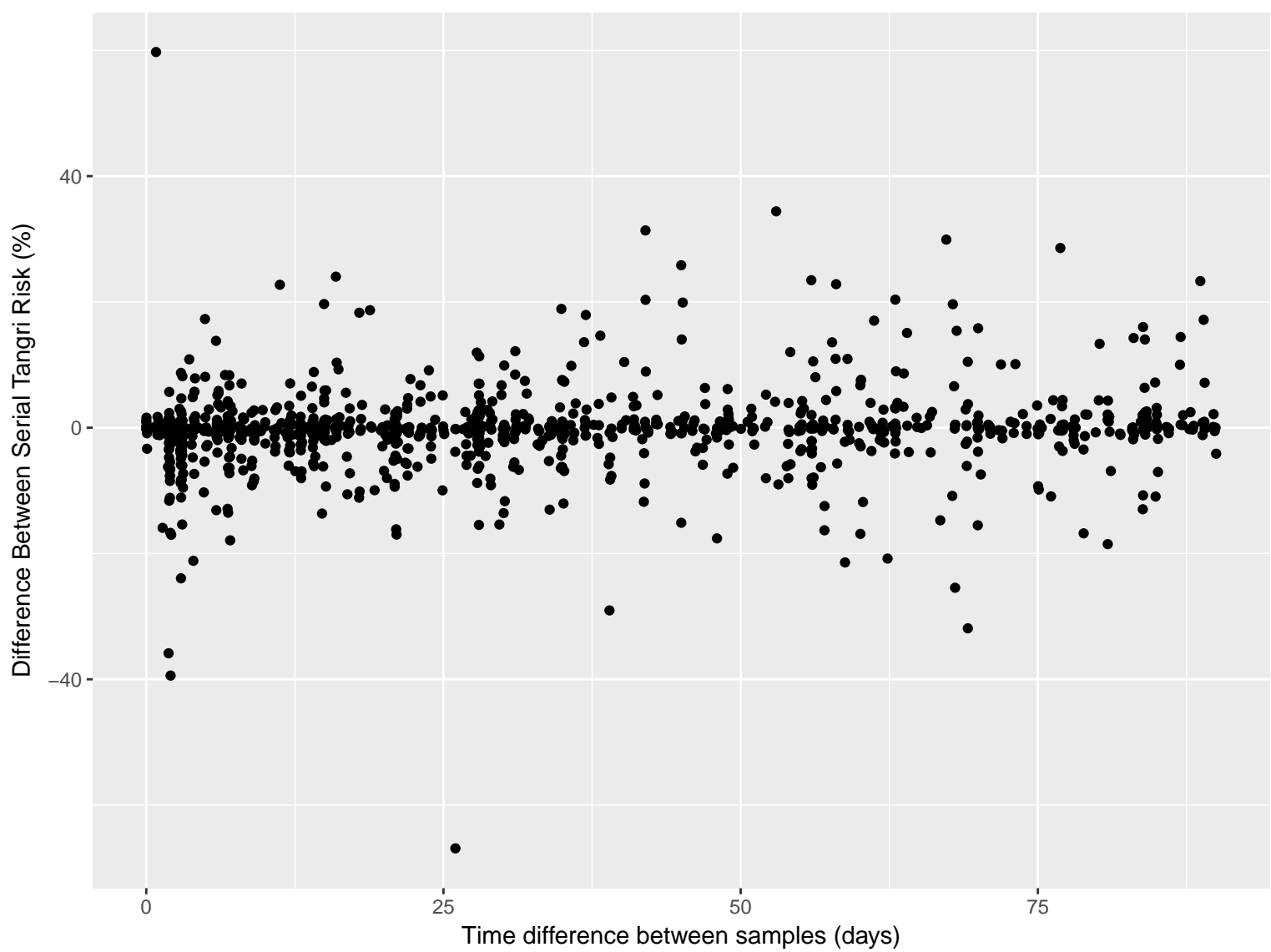

Supplement: S1 Fig — No signiciant difference was found between time periods of < 10 days and 10 days to 3 months (p = 0.16). (PDF) [file pone.0198456.s001.pdf]
