# Supplementary material for: Individual patient variability with the application of the kidney failure risk equation in advanced chronic kidney disease
Source: PLoS One. 2018 Jun 12;13(6):e0198456. doi: 10.1371/journal.pone.0198456 (PMC5997334; doi:10.1371/journal.pone.0198456)
Supplement: S1 Table — Note the point estimate of KFRE changes but variability not significantly different at the same KFRE. (DOCX) [file pone.0198456.s002.docx]

**Day-to-day variability of kidney failure risk with different ages in males and females.**

| Age in years | Sex | eGFR | ACR | KFRE point estimate | Variability range | variability |
| --- | --- | --- | --- | --- | --- | --- |
| 20 | Male | 25 | 30 | 30% | 25%-36% | 11% |
| 20 | Female | 25 | 30 | 24% | 20%-29% | 9% |
| 30 | Male | 25 | 30 | 25% | 20%-30% | 9% |
| 30 | Female | 25 | 30 | 20% | 17-24% | 7% |
| 40 | Male | 25 | 30 | 21% | 17%-25% | 8% |
| 40 | Female | 25 | 30 | 17% | 14%-20% | 6% |
| 50 | Male | 25 | 30 | 17% | 14%-21% | 7% |
| 50 | Female | 25 | 30 | 14% | 11%-16% | 5% |
| 60 | Male | 25 | 30 | 14% | 11%-17% | 6% |
| 60 | Female | 25 | 30 | 11% | 9%-13% | 4% |
| 70 | Male | 25 | 30 | 11% | 9%-14% | 5% |
| 70 | Female | 25 | 30 | 9% | 7%-11% | 4% |
| 80 | Male | 25 | 30 | 9% | 7%-11% | 4% |
| 80 | Female | 25 | 30 | 7% | 6%-9% | 3% |
| 90 | Male | 25 | 30 | 7% | 6%-9% | 3% |
| 90 | Female | 25 | 30 | 6% | 5%-7% | 2% |

Note the point estimate of KFRE changes but variability not significantly different at the same KFRE.
